# Markers of Hypoxia and Metabolism Correlate With Cell Differentiation in Retina and Lens Development

**DOI:** 10.3389/fopht.2022.867326

**Published:** 2022-04-22

**Authors:** Tom A. Gardiner, Tiarnan Branagh, Nuala Tipping, Denise M. McDonald

**Affiliations:** ^1^ Centre for Biomedical Sciences Education, School of Medicine Dentistry and Biomedical Sciences, Queen’s University Belfast, Belfast, United Kingdom; ^2^ Wellcome Wolfson Institute for Experimental Medicine, School of Medicine, Dentistry and Biomedical Sciences, Queen’s University Belfast, Belfast, United Kingdom

**Keywords:** retina, hypoxia, metabolism, mitochondria, HIF-1α, HIF-2α, AMPK, PGC-1α

## Abstract

Recent studies have provided novel insights of co-development of the neural and vascular elements of the retina. Knowledge of these relationships are crucial to understand the impact of therapeutic measures in Retinopathy of Prematurity (ROP). ROP is imposed by therapeutic oxygen upon immature retinal blood vessels and neural cells causing delayed development and vascular regression. However, the impact of hyperoxia on developing retinal neurons is less understood because some aspects of normal development remain unknown. The metabolic changes during differentiation of retinal progenitor cells to functional neurons is one such aspect. We correlated immunomarkers of hypoxia with markers of metabolic change in developing retinal neurons during the early postnatal period in mice. The same marker proteins were studied in secondary lens fiber differentiation at postnatal day-3 (P3). Nuclear localization of the oxygen-sensitive subunits of hypoxia inducible factor, HIF-1α and HIF-2α was correlated with increasing mitochondrial content in differentiating neurons. Nuclear HIF was also correlated with AMP-dependent protein kinase (AMPK), and the AMPK phosphorylation target PPAR-gamma coactivator-1alpha (PGC-1α), the principal regulator of mitochondrial biogenesis. Expression of AMPK, PGC1α and HIF-2α in secondary fiber differentiation was visible in each profile of the lens equator. Strong nuclear localization for all markers was present at the onset of secondary fiber differentiation, and reflected changes in size, mitochondrial content, and metabolism. We speculate that the ‘physiological hypoxia’ that drives retinal vascular development is cell-specific and reliant upon neuronal differentiation and mitochondrial biogenesis. We suggest that the onset of differentiation increases energy consumption that is detected by AMPK. In turn AMPK increases mitochondrial biogenesis *via* PGC-1α. Mitochondrial oxygen consumption may then create intracellular hypoxia that activates HIF. This progression is congruent with the expression of these markers in secondary lens fiber differentiation and nuclear localization of HIF-2α. Nuclear localization of HIF-1α and HIF-2α in the postnatal retina is less defined than in the lens as it may involve the remnant of HIF expression from the embryonic period that is sustained and increased by intracellular hypoxia caused by increasing mitochondrial oxygen consumption. This the first report of the involvement of HIF-2α, AMPK and PGC-1α in lens development.

## Introduction

Tissue hypoxia represents an important pathological factor in several retinal vascular diseases (1), including retinopathy of prematurity (ROP) (2). ROP is the chief cause of vision loss in the young and affects premature infants exposed to therapeutic hyperoxia as part of life-sustaining neonatal care (2). The retinal pathology in ROP has 2 main phases: phase-1 is due to exposure of the immature retina to therapeutic oxygen that switches off the hypoxia-dependent transcription of vascular endothelial growth factor (VEGF), the principle vascular growth/survival factor for vascular endothelial cells (3), causing cessation of blood vessel growth and regression of the formed vessels (4). Phase-2 is caused by reintroduction of the infants to room air at which time vascular insufficiency results in tissue hypoxia that drives pathological neovascularization through hypoxia inducible factor, the transcriptional activator of VEGF expression.

Seminal studies by Chan-Ling and Stone introduced the concept of “physiological hypoxia” as the driving force in normal retinal vascular development (5) and it is this state that is disrupted by the introduction of hyperoxia in ROP (4). The term physiological hypoxia suggests a tolerable oxygen deficit that occurs in the course of normal development and differentiates it from the more profound cytotoxic hypoxia that represents a pathological factor in the second phase of ROP. Added complexity in the pathogenesis of ROP arises from the fact that the vascular pathology occurs in the rapidly developing neural retina where VEGF exerts neuroprotective effects that complicate therapeutic measures aimed at its inhibition (6). As recent studies have revealed new levels of precision in neuro-vascular co-development in retina (7, 8), it is a priority to understand the metabolic changes occurring in retinal neurons, and the possible impact of therapeutic interventions.

Recent studies have highlighted the widespread expression of the hypoxia-inducible factors HIF-1α and HIF-2α in the normal development of the neural retina and shown important cell-specific expression of these players, both in differentiated neurons and retinal progenitor cells (8–10). These studies draw attention to the importance of other HIF-regulated genes in retinal physiology and pathology (11) and although specific HIF target genes (12), especially erythropoietin have been shown to exert powerful cytoprotective effects on both vascular and neural cells in models of ROP (13–16), less attention has been paid to the role of HIFs in neural differentiation and the associated metabolic changes (17).

In the present study, the importance of cell-specific metabolism in response to hypoxia was emphasized by an initial exploration of physiological hypoxia in the development of the deep vascular plexus of the retina. Studies by Usui et al. have demonstrated the importance of increased metabolic demand by the developing horizontal and amacrine cells, in induction of the deep and intermediate retinal capillary plexuses respectively (7). We questioned whether the hypoxia generated by such changes, coupled with increased oxygen consumption by the developing photoreceptor cells (18) would be measurable with the hypoxia-sensitive bioreductive drug pimonidazole (19). We and others have used pimonidazole (Hypoxyprobe) to map regional pathological hypoxia in models of retinal vascular disease (8, 10, 20–22). It was noted that immunostaining for the drug adducts was particularly strong in the retinal ganglion cells within the hypoxic inner retina in oxygen-induced retinopathy (22), and while this was attributed to the normally high metabolic activity of the ganglion cells, the phenomenon was not further investigated. Therefore, in the present work we exposed neonatal mice to pimonidazole on postnatal day-5 (P5), P9 and P11 and found that the highest deposition of the probe was in the ganglion and amacrine cells. Importantly, it was noted that the HP did not deposit in either the astrocytes or the undeveloped progenitor cells. This was in spite of the fact that these cells resided in close proximity to the strongly labelled ganglion and amacrine cells, with presumably similar access to local oxygen.

Immunostaining for HIF-1α in retinas of mice aged P1, P3, P5 and P9 again showed the strongest expression and nuclear localization in the developed ganglion and developing amacrine cells, as compared to the retinal progenitor cells. Although the majority of ganglion, amacrine and horizontal cells, along with the cone photoreceptors, are “born” (defined as the time that they undergo their final S-phase and exit the cell cycle) in the prenatal period, only the ganglion cells, and to a lesser extent, the amacrines show significant physical development at P0. We therefore hypothesized that increasing cell size and metabolic activity could be linked to HIF activity in the ganglion and amacrine cells in response to the increased energy demands of protein synthesis (23) and neural signaling. Differentiation from relatively quiescent progenitor cells to large metabolically active neurons requires a switch from dependence on glycolysis to oxidative phosphorylation (17) that should correlate with mitochondrial density (24). A paradigm linking increased mitochondrial biogenesis to HIF-1α activity under physiological conditions has been described in muscle and will be discussed below (25).

In the present study, as mitochondrial density correlated with the nuclear HIF-1α in neonatal retinal development, we reasoned that any change in metabolism that increases mitochondrial density probably requires participation by the central energy sensor and metabolic regulator AMP-dependent protein kinase (AMPK) (26, 27), and the master regulator of mitochondrial biogenesis, peroxisome proliferator-activated receptor gamma coactivator-1α (PGC-1α) (28, 29). Accordingly, we sought to localize these proteins in the developing retina between P1 and P9. We also examined the pattern of HIF-2α expression and nuclear localization of phosphorylated glycogen synthase (pGS) as an indicator of metabolic maturity in retinal neurons (30, 31).

To further validate our hypothesis that HIF expression may occur in cells experiencing a rapid increase in mitochondrial metabolism as a consequence of differentiation, we compared the markers used in the retinal study to their expression in secondary lens fiber development at P3. Elegant studies have progressively revealed the molecular cell biology of secondary lens fiber development and provide a highly characterized model for the investigation of cellular differentiation in specialized cells (32–35). We therefore employed postnatal secondary lens fiber differentiation to evaluate the metabolic markers used in our study of the developing retina, and to validate our antibodies in a tissue that had undergone the same preparation regime, but which expressed a distinctly different proteome (36). In this we hoped to exclude alternative tissue-specific uses of the marker proteins chosen for our analysis.

## Methods

### Experimental Animals

Litters of C57/BL6 wild-type (WT) mice were obtained from the Biological Services Unit of QUB. Groups of 3 pups between P5 and P11 were injected with the oxygen sensitive drug pimonidazole (Hypoxyprobe Inc., Burlington, Massachusetts) at a dose of 60mg/per kg, given by intraperitoneal injection 3 hours prior to culling with an overdose of Pentobarbital Sodium as previously described (22). Additional groups of 5 pups were culled at P1, P5 and P9 and the eyes processed for wax histology and histochemistry as described below. Single eyes from 3 of the P1 and 3 of the P9 animals were fixed and processed for electron microscopy as described below.

All procedures with living animals were carried out under the United Kingdom 1986 Animals Scientific Procedures Act with supervision by the British Home Office and conformed to the ARVO statement on the use of animals in ophthalmic and vision research.

### Tissue Processing

The eyes used for wax histology and immunohistochemistry were enucleated under terminal anesthesia and fixed in 4% paraformaldehyde in 0.1 M phosphate buffer at pH 7.4 for 4 hours at room temperature. Fixation was followed by several washes in phosphate-buffered saline (PBS) and storage overnight in PBS at 4°C prior to dehydration in a graded ethanol series and embedding in BioPlast Plus wax embedding medium. Histological Sections were cut at 5µm, mounted on Superfrost Plus cationically-coated glass slides, and dried overnight at 65°C.

### Electron Microscopy

The eyes used for electron microscopy were fixed in 2.5% glutaraldehyde in 0.1M cacodylate buffer, given several buffer washes and stored in cacodylate at 4°C. After dissection, the eyes were post-fixed in 1% osmium tetroxide in cacodylate, dehydrated in ethanol and embedded in modified Spurr’s resin (Polysciences Europe). Ultrathin sections were mounted on uncoated copper grids and stained with 1% uranyl acetate in 50% ethanol and Reynold’s lead citrate.

The sections were imaged with a JEOL JEM-1400plus transmission electron microscope at an accelerating voltage of 80kv.

### Heat-Induced Epitope Retrieval (HIER)

All tissue sections used for immunohistochemistry were subjected to HIER as follows: Sections were thoroughly dewaxed and rehydrated through descending concentrations of ethanol and washed in distilled water. Slides were placed in stainless steel racks and transferred to 0.05% citraconic anhydride (CA) in distilled water at pH 7.4 pre-heated to 98°C for 30 minutes. The temperature of the CA bath was then rapidly reduced by stepwise replacement with CA at room temperature until the temperature reached 30°C. The slides were then given exhaustive washing with distilled water prior to immunostaining.

NB* When added to water the CA does not completely dissolve until the pH has been raised above 6.0 with 1M sodium hydroxide. CA buffers strongly at around pH 6.0 but requires careful adjustment as the solution approaches pH 7.0.

### Immunohistochemical Staining

The eye sections were thoroughly washed in distilled water followed by three 5-minute washes in PBS+ 0.5% Triton-X. Blocking was performed with 2% normal goat serum in PBS-Triton for 30 minutes at room temperature. Sections were transferred directly from the block solution to primary antibodies diluted in the same blocking solution and stained overnight at 4°C in the primary antibodies. The primary antibodies employed in this study were anti-Hypoxyprobe (HP) (Rabbit monoclonal antibody from Hypoxyprobe, Burlington, Mass. USA) at a concentration of 1:100. The remainder of this list were all rabbit polyclonal antibodies: anti- HIF-1α, ab228649- AbCam, Cambridge, UK, at a dilution of 1:200; anti-HSP-60 cat# RPCA-HSP60 (from EnCor Biotechnology Inc., Gainesville, Fl, USA) at a concentration of 1:1000; anti- HIF-2α from Novus Bio NB100-122SS, at a concentration of 1:100; anti-pAMPK-α1 (Thr174) from Millipore-UK 09-290 at 1:200; anti-phospho-AMPK-β1 (Ser108) from Santa Cruz Biotech sc-33525 at 1:200; anti- PGC-1α from Novus Bio NBP1-04676 at 1:200; anti-phospho-glycogen synthase (Ser641/Ser645) from Merck Millipore Cat # 07-817 at 1:200; anti-calbindin (Chemicon) at 1:1000. The anti-HIF-1α antibody was validated using cobalt chloride treated HeLa cells for a positive control, as described in the supplementary data file.

**Figure 1:**
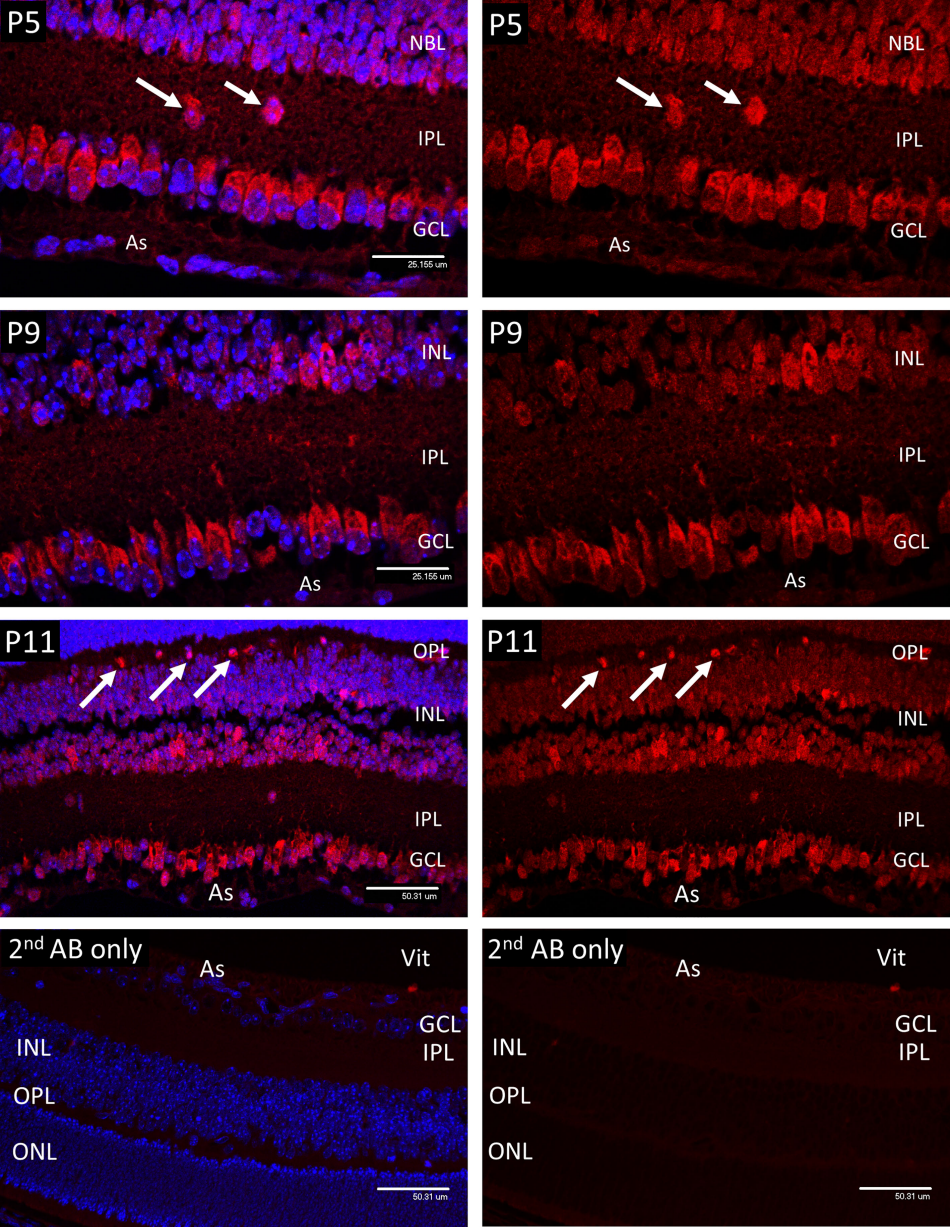
Confocal micrographs of Hypoxyprobe (HP) adducts (Red) in postnatal mouse retina. In P5 retina HP stain was only observed in the cytoplasm of the ganglion cells (GCL), developing amacrine cells at the inner aspect of the INL, and scattered cell bodies (arrows) withing the inner plexiform layer (IPL). No HP staining was present in the astrocytes (As). A similar pattern was noted in P9 retina, although by P11 staining for HP was evident in scattered cells at the outer edge of the INL (arrows); based on location these cells were presumed to represent horizontal cells. Ganglion cell layer (GCL), inner plexiform layer (IPL), inner nuclear layer (INL), outer plexiform layer (OPL). Vitreous body- Vit. Non-specific staining in the 2nd antibody controls was barely detectable.

Following staining with primary antibodies, the sections were washed extensively in PBS-Triton, blocked for 30mins, and transferred to Alexa-568 labelled anti-Rabbit polyclonal secondary antibody, Life Sciences UK at a dilution of 1:1000 for 1-2 hours at room temperature in the dark. After secondary antibody staining the sections were washed in PBS-Triton, counterstained in DAPI (0.01µg/ml in PBS) and mounted in Vectashield (Vector Laboratories Ltd.). Omission of the primary antibody served as a control for specificity of the secondary antibody (**Figure 1**), while the different staining patterns obtained with the various rabbit IgGs, combined with reference to previous studies, provided some internal assurance of the specificity of each.

### Confocal Microscopy

The stained sections were viewed with an Andor DSD spinning disc confocal system, fitted to an Olympus BX60 epifluorescence microscope. An Andor AMH metal halide light source was employed for confocal illumination, and images were collected using Andor iQ3 acquisition software.

## Results

### Hypoxyprobe Adducts in Postnatal Retinal Development

In the retina of P5 mice, HP adducts (**Figure 1**) were only consistently present in the cytoplasm of the ganglion cells (GCL). HP staining was also observed in a scattered population of cells considered to be developing amacrine cells at the inner aspect of the internal nuclear layer (INL), also in occasional cell bodies within the inner plexiform layer (IPL). HP staining was not observed in astrocytes (As) at any stage. A similar pattern was noted in P9 retina, although by P11 staining for HP was evident in scattered cells at the outer edge of the INL (arrows) that were presumed to be horizontal cells, based on their location.

### Nuclear HIF-1α in Postnatal Retinal Development

**Figure 2:**
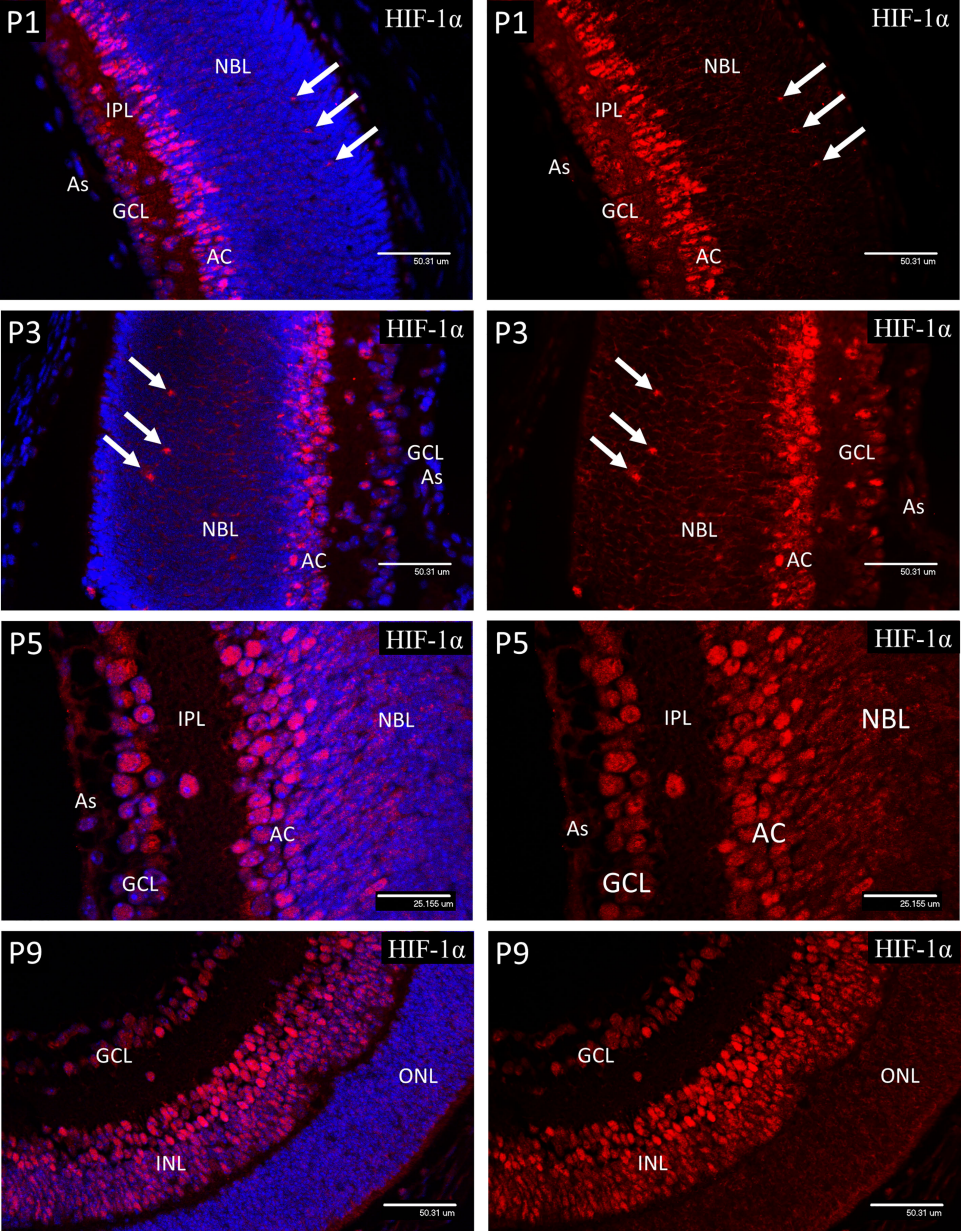
Confocal micrographs showing immunostaining for HIF-1α in postnatal mouse retina (Red). In P1 retina nuclear staining for HIF-1α was present in ganglion cells (GCL) and developing amacrine cells (AC) at the inner aspect of the neuroblastic layer (NBL). Staining was also detectable in nuclei at the level of the developing horizontal cells (arrows). A similar pattern was noted at P3, but with increased staining in the presumptive horizontal cells (arrows) at the outer region of the NBL. At P5, nuclear staining for HIF-1α was present in the majority of cells in the GCL, cells in the IPL and in the developing amacrine cells at the inner margin of the INL. In P9 retina the majority of the cells in the INL (arrows) showed nuclear staining for HIF-1α, although there was a gradient in staining intensity, increasing from the outer to inner aspect. There was also a reduction in nuclear HIF-1α staining in the majority of the ganglion cells (GCL) at P9. No nuclear HIF-1α staining was observed in astrocytes (As) at any stage.

In P1 retina nuclear HIF-1α staining (**Figure 2**) was present in ganglion cells (GCL) and developing amacrine cells (AC) at the inner aspect of the neuroblastic cell layer (NBL). Staining was also detectable in nuclei at the level of the developing horizontal cells in the outer third of the NBL. A similar pattern was noted at P3, but with increased staining in the presumptive horizontal cells (HZ). At P5 nuclear HIF-1α staining reflected the HP deposition observed for HP deposition at the same time point with strong nuclear staining for HIF-1α present in the majority of cells in the GCL, cells in the IPL and in the developing amacrine cells at the inner margin of the INL. In P9 retina the majority of the cells in the INL showed various levels of nuclear staining for HIF-1α, but with an obvious gradient from the outer to inner aspects, being strongest in the amacrine cells at the inner margin, possibly as a result increasing oxygen tension due to the developing deep vascular plexus at this time (37). Likewise, HIF-1α staining was reduced in the majority of the ganglion cells (GCL) reflecting the presence of the maturing primary vascular plexus. No nuclear HIF-1α staining was observed in astrocytes (As) at any stage.

### Calbindin Staining for Identification of Neuronal Subtypes

**Figure 3:**
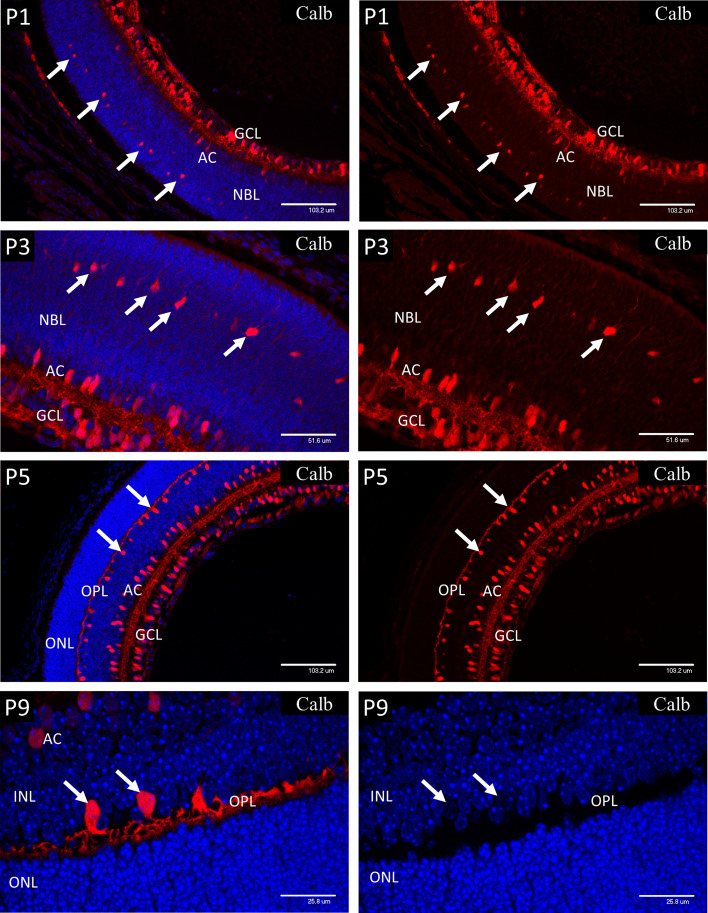
Postnatal mouse retina immunostained for calbindin. At P1, strong immunostaining for Calbindin is present in the ganglion (GCL) and amacrine cells (AC). Calbindin also marks the developing horizontal cells (HZ) in the outer third of the NBL (arrows). At P3, calbindin staining showed enlargement of the HZ cells and revealed extension of fine neurites orientated toward the outer retina (arrows). At P5 the OPL is outlined by the processes of the HZ cells with their cell bodies located in annexes of the OPL at the outer margin of the INL. P9 retina shows further development of the HZ cell processes in the OPL and the precise location of their cell bodies (left P9 image). The HZ cell nuclei stained extremely weakly with DAPI suggesting a high proportion of euchromatin (arrows-P9 right side image).

The calcium-binding protein calbindin (**Figure 3**) is widely employed as a marker of retinal neurons and is regarded as specific for horizontal cells (HZ), even though it also stains amacrine and ganglion cells. In retinal sections, this differentiation is regarded as unambiguous since the HZ cells reside in a morphologically defined layer of the retina that is widely separated from the amacrines and ganglion cells. Specifically, in adult retina the HZ cells occupy the outer margin of the inner nuclear layer, with their cell bodies located in annexes of the outer plexiform layer (OPL). During development the OPL is defined by the horizontal processes of the HZ cells from P5 at the time of cleavage of the NBL to produce the OPL and INL. However, the HZ cell bodies reside in a regular layer at the same location in the outer third of the NBL prior to lamination at the ONL. This layer was revealed in sections of P1 and P3 retinas stained for calbindin (**Figure 3**) and provides a positive correlation between HZ cells and the cells of the outer NBL that stained positively for nuclear HIF-1α, and the other markers described below. At P1, strong immunostaining for calbindin was present in the ganglion (GCL) and amacrine cells (AC) and the developing horizontal cells in the outer third of the NBL (arrows). At P3, calbindin staining revealed enlargement of the HZ cell cytoplasm and extension of fine neurites orientated toward the outer retina. From P5 the OPL is outlined by the processes of the HZ cells with their cell bodies located in annexes of the OPL at the outer margin of the INL (see P9 images of **Figure 3**). The cell nuclei of the HZ cells stained extremely weakly with DAPI at all stages, suggesting a high proportion of euchromatin. This was confirmed by electron microscopy. The typical morphology and location of the HZ prior to P5 does not appear to be well appreciated, therefore we have provided further details and conventional brightfield images in the supplementary data (**Figure S2**).

### Distribution of Mitochondria in the Developing Retina

At P1 and P3, HSP-60 immunostaining was confined to brightly stained sub-micron bodies, assumed to be mitochondria, that were abundant in the GCL and developing amacrine cells, but were fewer in the astrocytes and had only a sparse distribution throughout the NBL. However, mitochondria were present in a regular organized fringe among the dividing cells of the ventricular zone at the outer edge of the NBL, corresponding to early photoreceptor inner segments (**Figure 3**).

**Figure 4:**
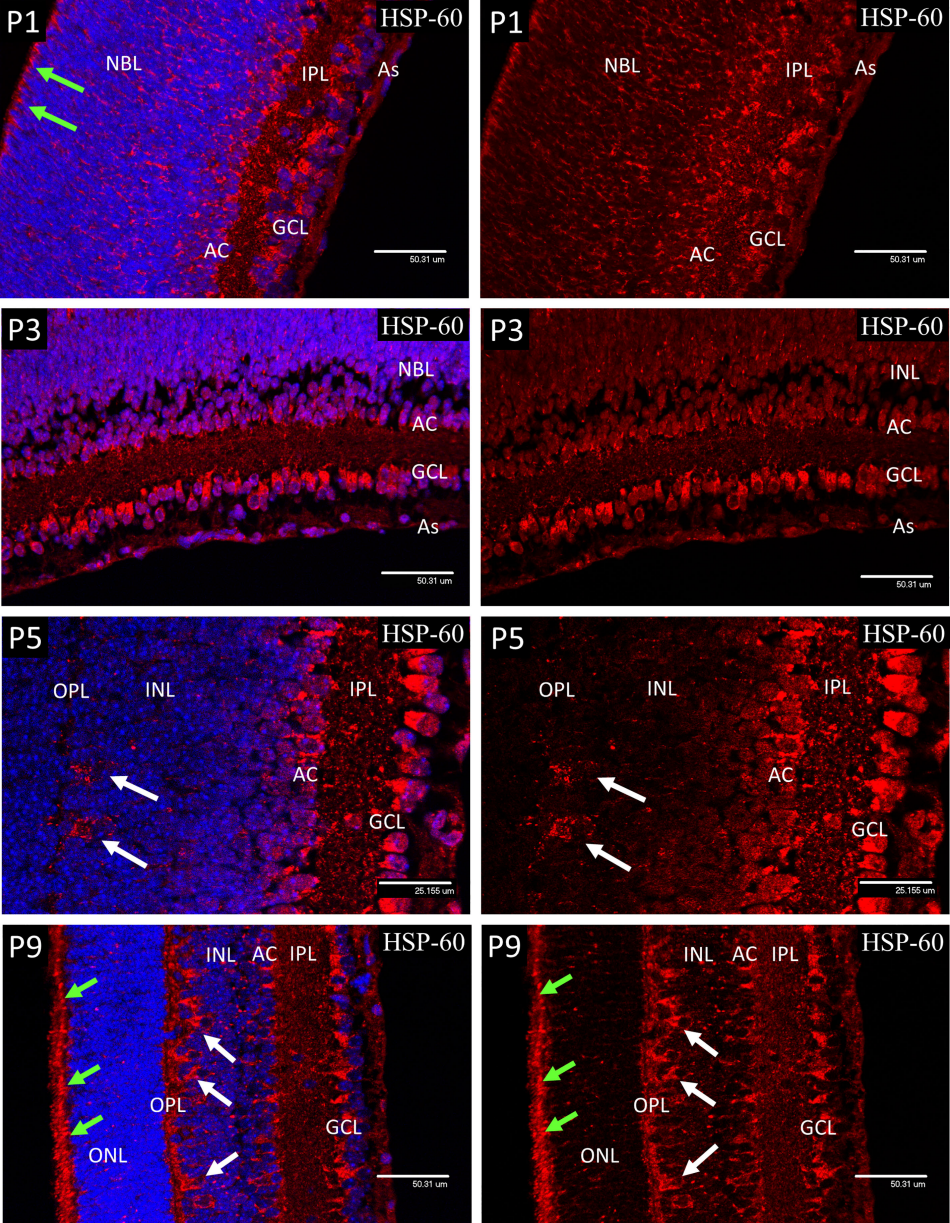
Confocal micrographs of postnatal mouse retina showing mitochondrial staining with anti-Hsp60 (Red). At P1 and P3 the mitochondria appeared as dense accumulations of brightly stained sub-micron bodies in the ganglion cells (GCL), developing amacrine cells (AC) and in the cells of the ventricular zone (arrows), but were fewer in the astrocytes and sparse in the NBL. A similar pattern was noted at P5 but included numerous mitochondria in the enlarging horizontal cells and their processes (arrows) at the interface of the INL and the developing outer plexiform layer (OPL). At P9 the mitochondrial density had greatly increased in the horizontal cell bodies (white arrows) and in the adjacent outer plexiform layer (OPL), presumably in HZ cell processes and photoreceptor synapses. The regular border of mitochondria at the outer edge of the neural retina had also become denser with increased development of the photoreceptor inner segments (green arrows).

**Figure 5:**
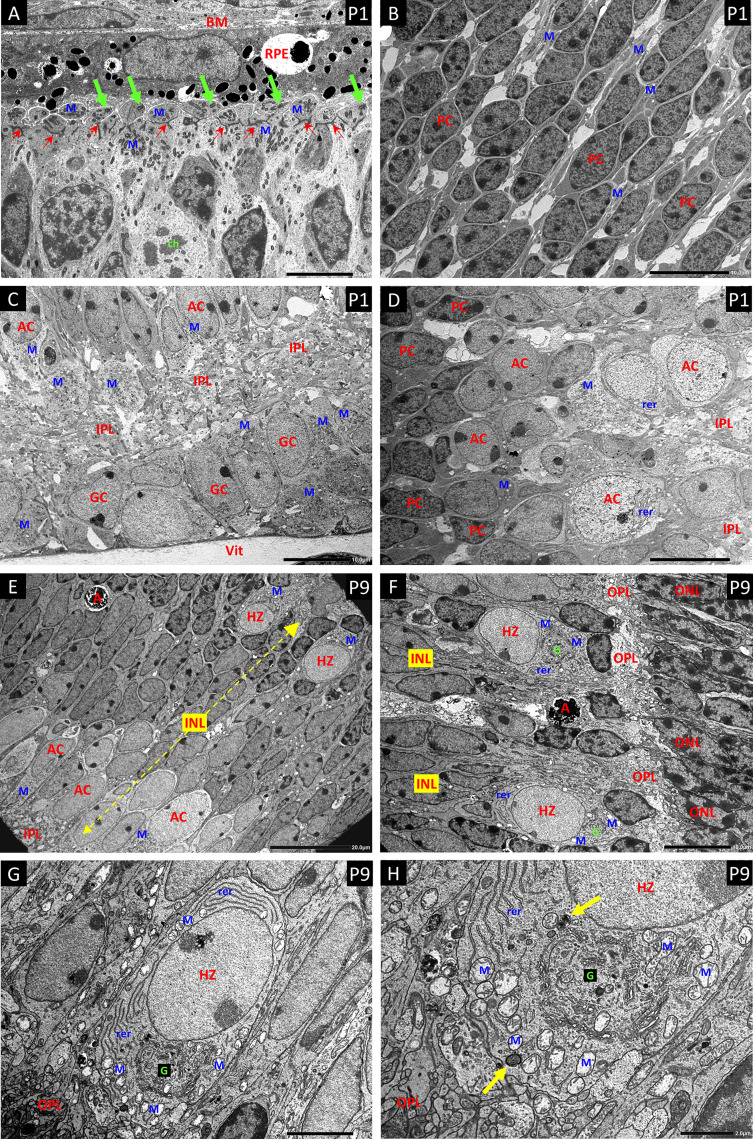
Electron micrographs (EM) of P1 retina **(A–D)** and P9 retina **(E–H)**. **(A)** The ciliated cells of the ventricular zone that give rise to the photoreceptors show large aggregations of small darkly stained mitochondria (M) distributed at both the inner and outer aspects of the layer of junctional complexes that will further develop into the external limiting membrane of the retina (Small red arrows). The outer portions are in close apposition to the retinal pigment epithelium (RPE) and their apical plasma membranes show the basal bodies and short shafts of sensory cilia (green arrows). Bruch’s membrane – BM. A dividing cell shows condensed chromosomal bodies (ch). **(B)** The progenitor cells (PC) of the central NBL show nuclei with a high proportion of dense heterochromatin, and sparse cytoplasm with only scattered mitochondria (M) as seen with HSP-60 staining. **(C)** EM of the innermost retina shows large aggregates of pale mitochondria (M) in the ganglion cells (GC) and developing amacrine cells (AC) at the inner border of the NBL. Developing inner plexiform layer – IPL; vitreous body - Vit. **(D)** Panel-D shows the gradation from heterochromatic PCs at the left, through an intermediate stage with a high proportion of euchromatin but little cytoplasm or organelles, to more typical amacrine cells (AC) with abundant cytoplasm replete with mitochondria (M) and rough endoplasmic reticulum (rer) at the edge of the IPL. **(E)** EM a full thickness profile of the inner nuclear layer at P9 shows mitochondria rich ACs and HZs at the inner and outer margins respectively. Apoptotic cells (A) are common in the INL at P9. **(F)** EM showing typical HZ cells at the OPL. They have distinctive euchromatic nuclei and cell bodies that occupy annexes of the OPL. All the HZ cells at P9 showed a high content of rough endoplasmic reticulin (rer) at various aspects of the nucleus; however, the extensive Golgi apparatus G was typically located toward the OPL. **(G, H)** Higher magnification EMs show mitochondria (M) at all aspects of the nucleus, but clustered at the apical border with the OPL, outboard of the highly developed Golgi complex G. Mitophagy-related bodies are located adjacent to intact mitochondria (yellow arrows).

At P5 both ganglion and amacrine cells again showed a high mitochondrial density, so that in places it was difficult to resolve individual organelles (**Figure 4**). Also, at P5, numerous mitochondria were present in the enlarging horizontal cells (HZ) and their processes at the interface of the inner nuclear layer (INL) and the developing outer plexiform layer (**Figure 4**). In P9 retinas, as at P1-5, large numbers of mitochondria were present in the ganglion cells and developing amacrine cells. Also, at P9 the mitochondrial density had greatly increased in the horizontal cell bodies (**Figure 4**, white arrows) and in the adjacent outer plexiform layer (OPL), presumably in neural processes and synapses (**Figure 4**), although by P9 some retinal microglia will have reached the OPL and will be responsible for a small component of the HSP-60 staining in that layer. The regular border of mitochondria at the outer edge of the neural retina had also become denser with increased development of the photoreceptor cells (**Figure 4**, green arrows). The distribution of mitochondria observed with HSP-60 immunostaining was confirmed in P1 and P9 eyes using electron microscopy (**Figure 5**).

Electron Microscopy of P1 (**Figures 5A–D**) retinas confirmed the distribution of mitochondria observed by HSP-60 immunostaining with high concentrations in the ganglion cells and amacrine cells at the inner margin of the NBL. Large concentrations of small dense mitochondria were also present in the ciliated cells at the outer margin of the NBL, on both inner and outer sides of the junctional layer that represents the precursor of the external limiting membrane of the adult retina.

In P9 retina (**Figures 5E–H**), electron microscopy not only confirmed the high concentration of mitochondria in the HZ cells, but also revealed extensive development of the rough endoplasmic reticulum and Golgi apparatus. Indeed, the density and development of these organelles in HZ cells at P9 (**Figures 5G, H**) exceeded that in amacrine cells at P9 and was comparable to that in the large amacrine cells in adult mouse retina. Electron microscopy also showed that the HZ cell nuclei were typically extremely euchromatic and explained the weak DAPI signal in fluorescent staining of these cells (see DAPI stain in P9 image of **Figure 3**). Many of the mitochondria in the inner retinal neurons in these specimens showed artefactual swelling that could have been due to fixative composition. However, as the eyes were fixed whole to maintain the structural integrity of the retina, the short fixation delay could also have been responsible as glutaraldehyde penetrates much more slowly than formalin.

### Nuclear HIF-2α in Postnatal Retinal Development

**Figure 6:**
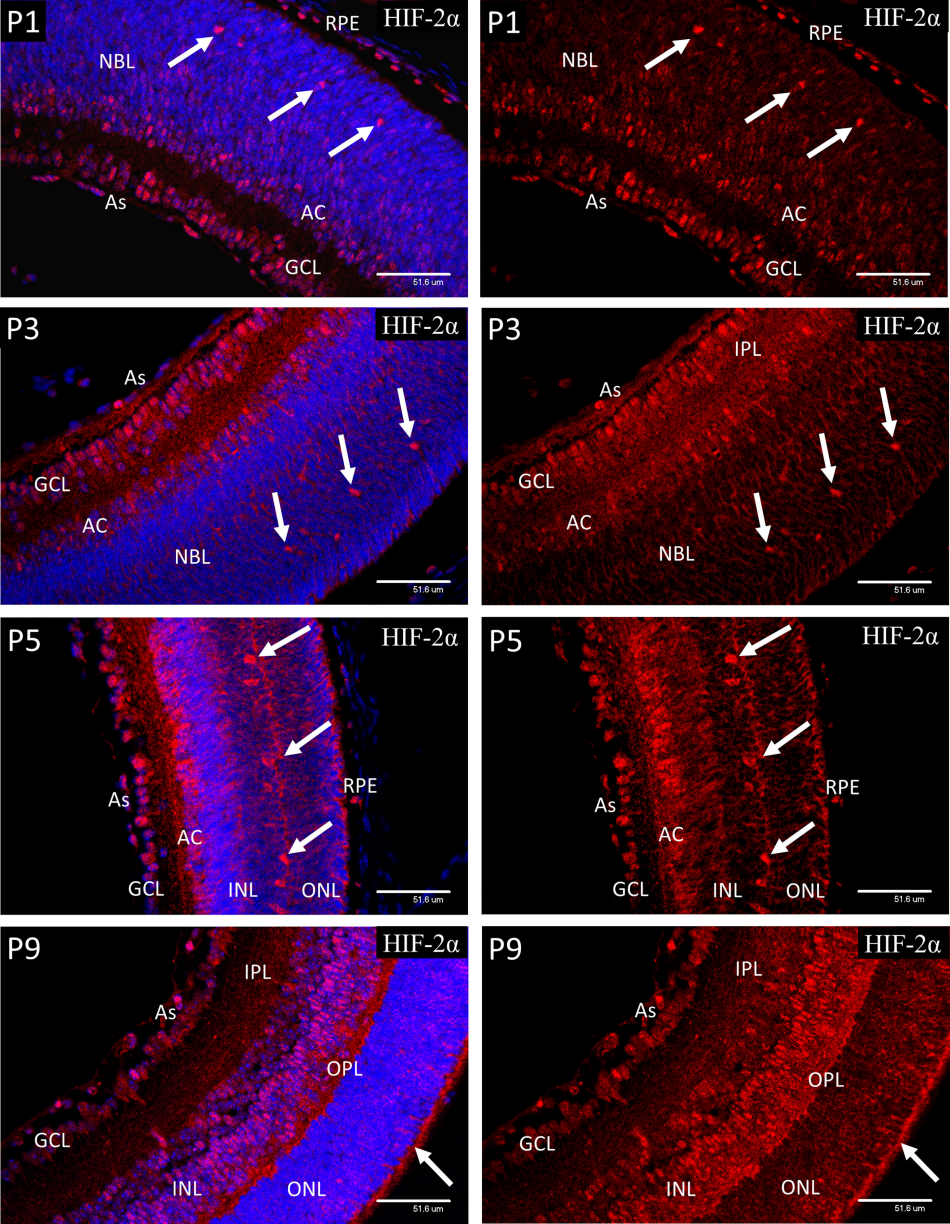
Confocal micrographs showing HIF-2α immunostaining in postnatal mouse retina (red). In P1 retina strong nuclear staining for HIF-2α was present in the astrocytes, ganglion cells (GCL), developing amacrine cells (AC) at the inner aspect of the NBL and the presumptive horizontal cells in the outer portion of the NBL. Strong nuclear staining was also present in the RPE. Weaker nuclear staining was also present in many progenitor cells in the NBL. A similar pattern was noted at P3, but nuclear localization was reduced in the amacrine, and NBL layers compared to the horizontal cells (arrows). A similar pattern was noted at P3, but nuclear localization was reduced in the amacrine, and NBL compared to the horizontal cells. Increased cytoplasmic staining was obvious in the neural processes of the IPL and a band within the NBL. At P5 the staining pattern was similar to P3 but by this time only occasional RPE cells showed nuclear localization if HIF-2α. At P9 a significant reduction was observed in nuclear HIF-2α in the GCL, as compared to P5. The majority of the cells in the INL at P9 showed nuclear staining. Also, at P9 strong cytoplasmic staining was present in the developing outer plexiform layer (OPL) and photoreceptor inner segments (arrows). Astrocytes at P9 continued to show strong nuclear localization of HIF-2α (As).

In P1 retina strong nuclear staining for HIF-2α was present in the astrocytes, ganglion cells (GCL), developing amacrine cells (AC) at the inner aspect of NBL and the presumptive horizontal cells in the outer third of the NBL (**Figure 6**). Strong nuclear staining was also present in the RPE. Weaker but definite nuclear staining was also present in many progenitor cells in the NBL and contrasted to the results for HIF-1α. A similar pattern was noted at P3, but nuclear localization was reduced in the amacrine, compared to the horizontal cells. Also at P3, increased cytoplasmic staining was obvious in the neural processes of the IPL. At P5 the staining pattern was similar to P3 but by this time only occasional RPE cells showed nuclear localization of HIF-2α. At P9 a significant reduction was observed in nuclear HIF-2α in the ganglion cells, as compared to P5, while at P9 the majority of the cells in the INL showed nuclear localization. Also, at P9 strong cytoplasmic staining was present in the developing outer plexiform layer and photoreceptor inner segments. As at all earlier stages, the astrocytes showed strong nuclear localization of HIF-2α.

### AMPK Expression in Postnatal Retinal Development

Antibodies to AMPK isoforms carrying the most common activating phosphorylations were chosen for the present study (38). Antibodies targeting the activating Thr174 phosphorylation site on the catalytic subunit of AMPK-α1 and the activating Ser108 phosphorylation site on AMPK-β1 were chosen as the mouse retina was reported to show similar expression of the alpha-1 and alpha-2 isoforms, but had a strong bias for the beta-1 subunit (39).

**Figure 7:**
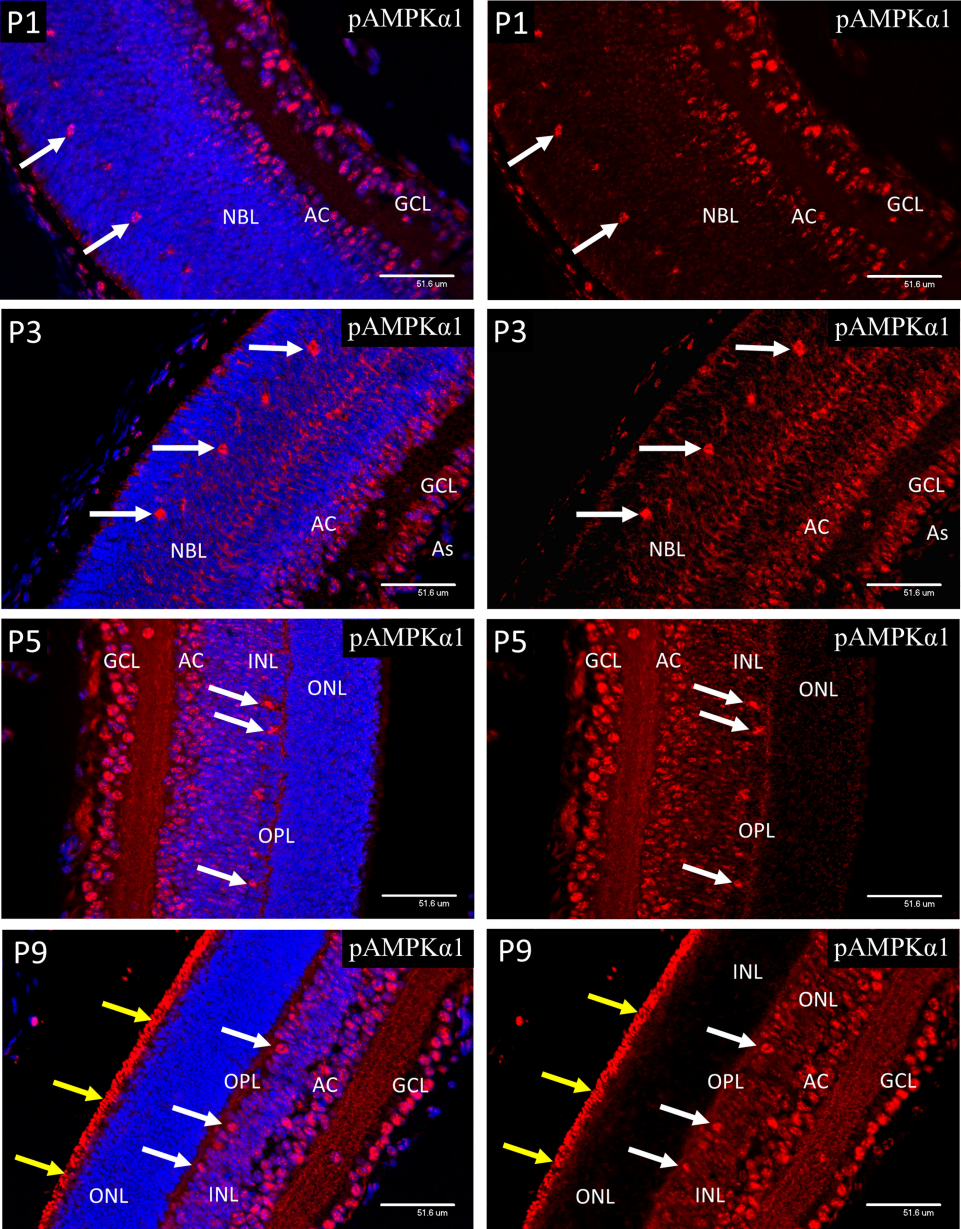
Confocal micrographs of postnatal mouse retina immunostained for pAMPKα1 (**Figure 7**). In P1 retina strong nuclear staining for pAMPKα1 was present in ganglion cells (GCL), developing amacrine cells (AC) and horizontal cells (white arrows). A similar pattern of nuclear staining was noted at P3, but cytoplasmic staining had increased in all retinal layers, revealed in the horizontal cells by positive staining of the enlarging cytoplasm and neurites. At P5 the ganglion, amacrine and horizontal cells continued to show strong nuclear staining for pAMPKα1, and positive staining was present in the neural processes of the developing outer plexiform layer (OPL). At P9 strong nuclear staining for pAMPKα1 was present in the ganglion, amacrine and HZ cells, as noted at P5. However, strong staining was also present in the newly developed photoreceptor outer segments (yellow arrows), which were not present at P5.

**Figure 8:**
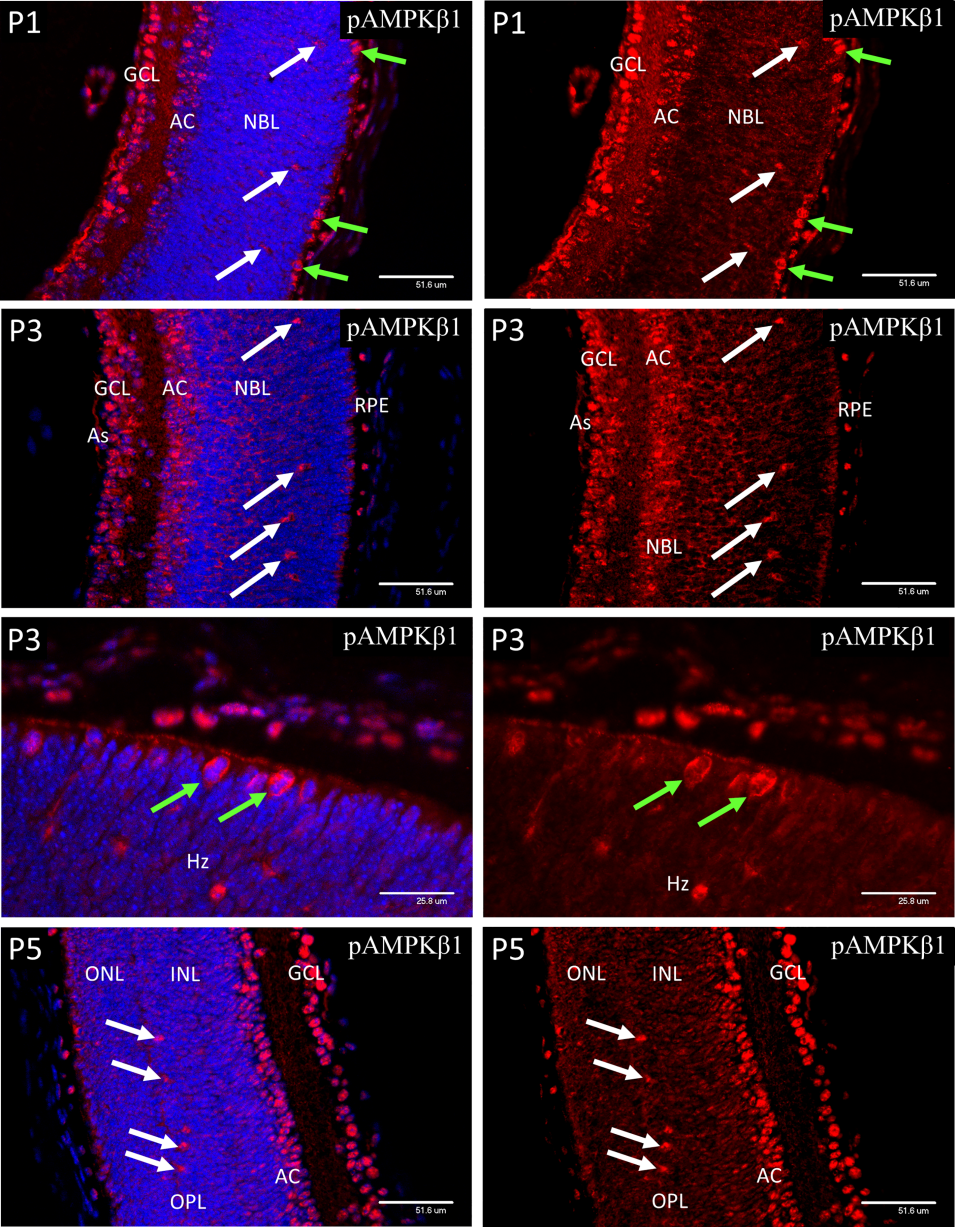
Confocal micrographs of postnatal mouse retina immunostained for pAMPKβ1. In P1 retina strong nuclear staining for pAMPKα1 was present in ganglion cells (GCL), developing amacrine cells (AC) and horizontal cells (white arrows). A similar pattern of nuclear staining was noted at P3, but cytoplasmic staining had increased in all retinal layers, revealed in the horizontal cells by positive staining of the enlarging cytoplasm and neurites. At P5 the ganglion, amacrine and horizontal cells continued to show strong nuclear staining for pAMPKβ1. Note that the dividing progenitor cells at the ventricular zone of the NBL stained more strongly for the phosphorylated beta subunit (**Figure 8**, green arrows in P3 images).

At P1, both pAMPK-1α (**Figure 7**) and pAMPK-1β (**Figure 8**) showed mainly nuclear staining, in a closely similar pattern to the HIFs, although at P3 there was a noticeable increase in cytoplasmic staining in the progenitor cell layer. At P5 nuclear staining had increased in the progenitor cells lying at the central region of the INL, although the most intense staining was still in the GCL, amacrine cells (AC) and horizontal cells. The only significant difference in localization between the 2 different activated isoforms of AMPK throughout the neonatal period was that pAMPK-1β (**Figure 8**) showed stronger staining in the dividing cells of the ventricular zone and pAMPK-1α (**Figure 7**) staining was especially strong in the developing photoreceptor outer segments (POS) at P9. This is in keeping with the multiple functions fulfilled by AMPK in dividing cells (40–44), although it is unclear why phosphorylation of the regulatory beta-subunit at Ser-108 should be particularly prominent, likewise the strong pAMPK-1α stain in the photoreceptor outer segments at P9. Such differential staining of the AMPK subunits may suggest different subunit combinations or simply relate to relative exposures of the different epitopes in particular situations. **Figure 8** shows a high magnification image of mitotic cells at P3, but the same phenomenon was present in all mitotic figures observed between P1 and P5.

### PGC-1α Localization in Postnatal and Adult Murine Retina

**Figure 9:**
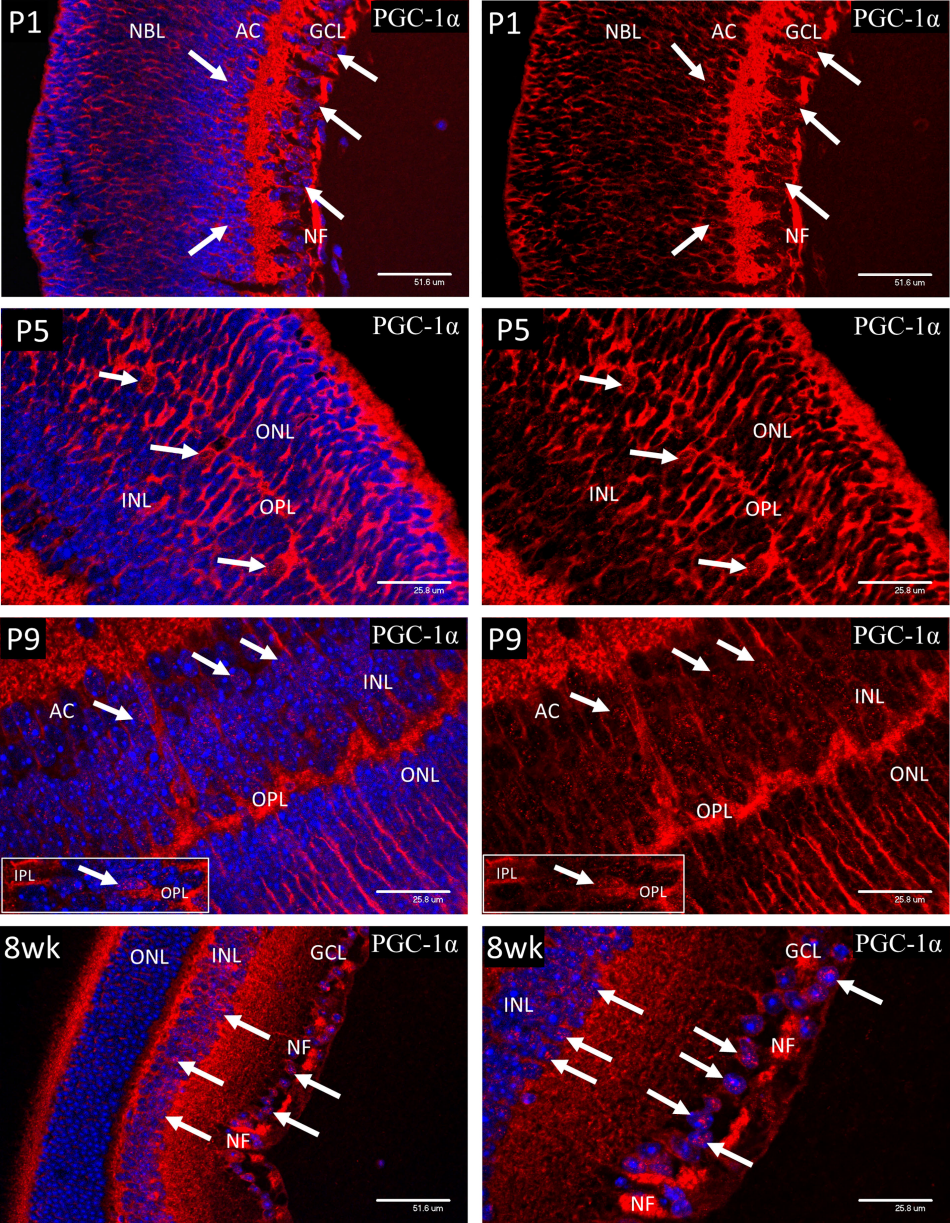
Cytoplasmic staining for PGC-1α was present throughout the retina from P1 to P9 and was particularly strong in the nerve fibers of the IPL and ganglion cell axons of the nerve fiber layer (NF). Initially, nuclear localization of PGC-1α (white arrows) was only notable in the ganglion and developing amacrine cells at P1-3 and horizontal cells at P5. There was a general increase in number of cells showing nuclear localization throughout the INL after P5 and by P9 the majority of the cells in GCL and INL showed positive nuclear staining, which is the adult pattern shown at 8 weeks of age. Occasional cell nuclei at P9 showed unusually strong nuclear staining. These nuclei lay in the central region of the INL, corresponding to the location of Muller cell bodies (P9 insert). The strong staining of the GC axons in the nerve fiber layer (NF), noted from P1 persisted into adulthood at 8-weeks. Interestingly, nuclear localization of PGC-1α was not commonly observed in the photoreceptor cells, either during development or in the mature retina at 8-weeks.

Cytoplasmic staining for PGC-1α (**Figure 9**) was present throughout the retina from P1 to P9 and was particularly strong in the nerve fibers of the IPL and ganglion cell axons of the nerve fiber layer (NF). However, nuclear localization of PGC-1α tended to be sporadic and was only widespread in the ganglion and developing amacrine cells at P1-3 and horizontal cells at P5. The number of cells showing nuclear localization increased from P5 and by P9 the majority of the cells in GCL and INL showed positive nuclear staining, which is the adult pattern shown at 8 weeks of age. Occasional cell nuclei at P9 showed unusually strong nuclear staining. These nuclei lay in the central region of the INL, corresponding to the location of Muller cell bodies (P9 insert). The strong staining of the GC axons in the nerve fiber layer (NF), noted from P1 persisted into adulthood. Interestingly, nuclear localization of PGC-1α was rarely observed in the photoreceptor cells after P5 or in the mature retina at 8-weeks.

### Phospho-Glycogen Synthase in Neonatal and Adult Murine Retina

**Figure 10:**
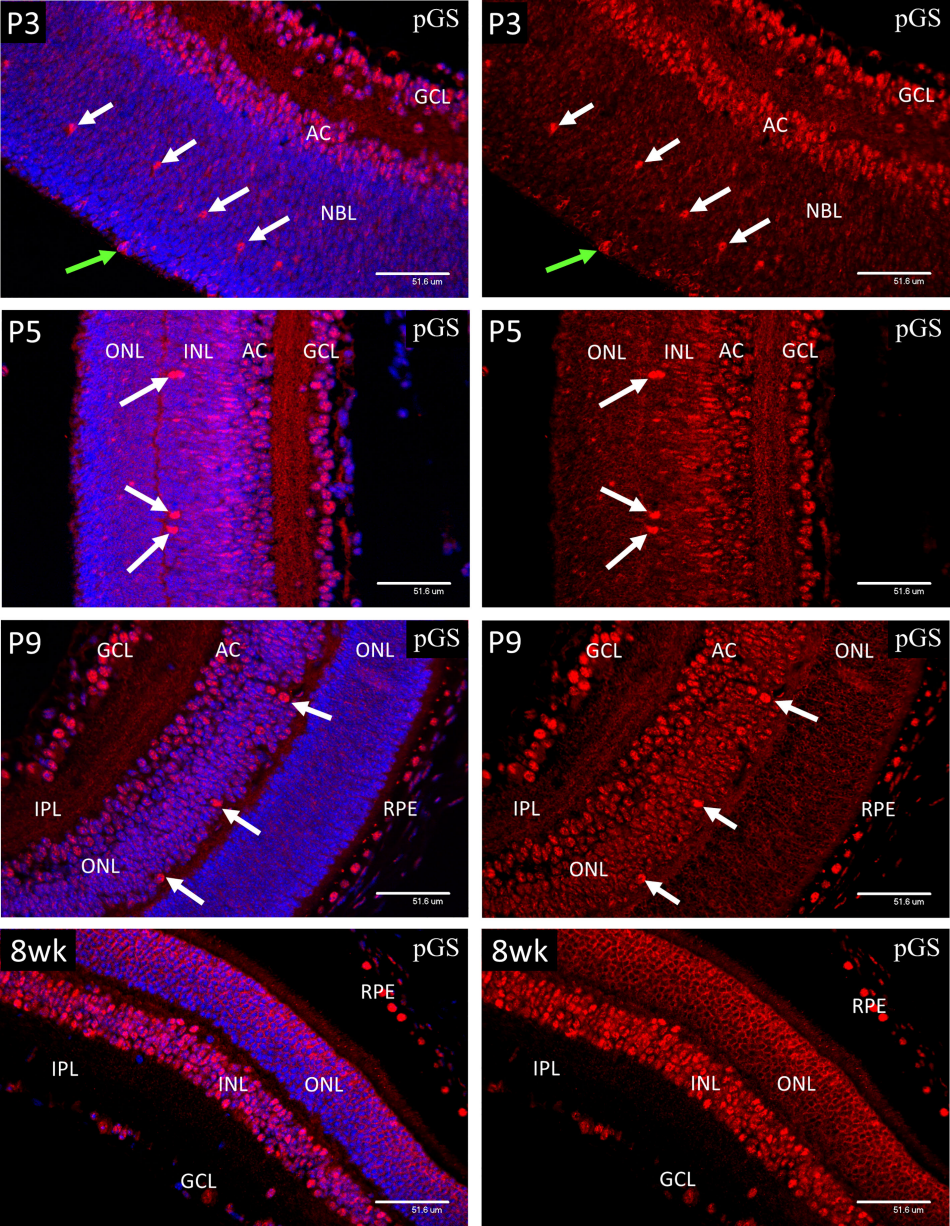
Confocal micrographs showing immunostaining for phospho-glycogen synthase (pGS) in postnatal mouse retina (Red). In P1-P3 retina, strong nuclear staining for pGS was present in ganglion cells (GCL), developing amacrine (AC) and horizontal cells (arrows), and mitotic cells of the ventricular zone (green arrows). In P9 retina, the majority of the cells in the INL showed an increase in nuclear pGS, although the photoreceptor cells were yet to develop the adult pattern as shown in images of 8-week (8wk) retina. Note that the chicken-wire appearance of the ONL is because pGS, when sequestered in the nucleus, occupies the euchromatin, which in rod photoreceptors is located at the nuclear periphery. Nuclear localization of pGS was observed in the RPE at all stages examined.

In P1-P3 retina, strong nuclear staining for pGS (**Figure 10**) was present in ganglion cells (GCL), developing amacrine (AC) and horizontal cells (arrows), and mitotic cells of the ventricular zone (green arrows). In P9 retina, the majority of the cells in the INL showed an increase in nuclear pGS, although the ganglion, amacrine and HZ cells still showed more nuclear pGS than those with cell nuclei in the central region of the INL. The photoreceptor cells at P9 were yet to develop the adult pattern as shown in images of 8-week (8wk) retina. Note that the chicken-wire appearance of the ONL at 8-weeks is because pGS, when sequestered in the nucleus, occupies the euchromatin, which in rod photoreceptors is located at the nuclear periphery. Nuclear localization of pGS was observed in the RPE at all stages examined.

### Expression of Metabolic Markers in Secondary Lens Fiber Development

**Figure 11:**
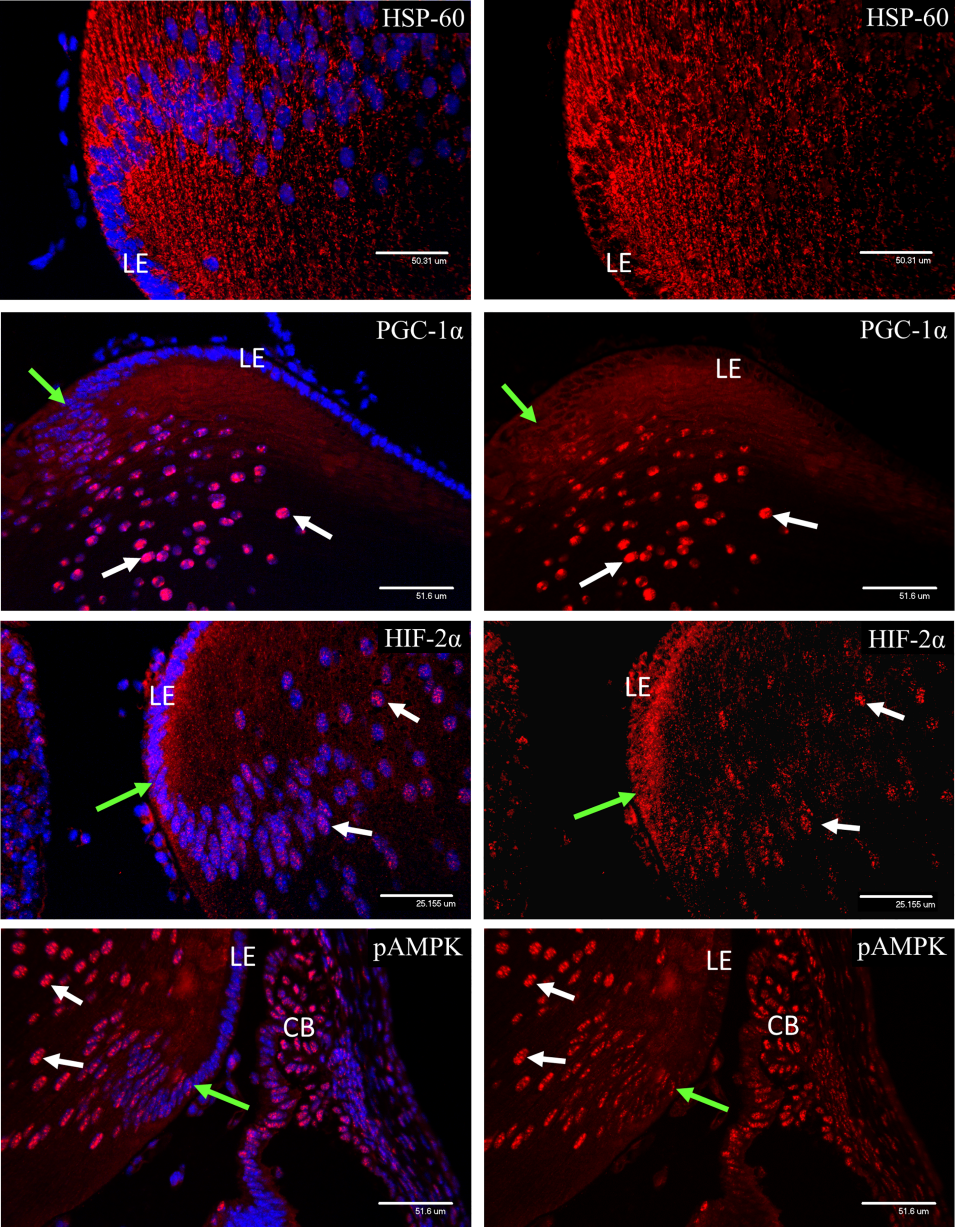
Confocal micrographs of P3 lens, showing nuclear localization of the metabolic markers observed in the retina. HSP-60 staining revealed a rich endowment of mitochondria in the lens at P3. PGC-1α was not detectable in the lens epithelium but appeared soon after elongation of the secondary lens fibers had commenced (green arrows) and showed strong nuclear localization (white arrows). HIF-2α was present in the cytoplasm of the lens epithelium at the equator of the lens, but nuclear localization (white arrows) was only observed after the point where secondary fiber development had commenced (green arrows). Both phosphorylated isoforms of AMPK showed weak staining in the lens epithelium, that increased and showed strong nuclear localization (white arrows) following the start of secondary fiber elongation (green arrows).

HSP-60 staining revealed a rich endowment of mitochondria throughout the secondary lens fibers at P3 (**Figure 11**). PGC-1α was not detectable in the lens epithelium but appeared soon after elongation of the secondary lens fibers had commenced and showed strong nuclear localization. No nuclear localization of HIF-1α was detected in the lens at P3 (data not shown). In contrast, HIF-2α was present in the cytoplasm of the lens epithelium at the equator of the lens, and nuclear localization increased after the point at which secondary fiber development had commenced (**Figure 11**). Both phosphorylated isoforms of AMPK showed weak staining in the lens epithelium, that increased and showed strong nuclear localization following the start of secondary fiber elongation (**Figure 11**).

## Discussion

This study has shown that cellular deposition of the oxygen sensitive drug pimonidazole/Hypoxyprobe (HP) in the developing retina is related to the mitochondrial density and that the drug did not readily mark cells whose metabolism was more dependent on glycolysis, such as astrocytes and Muller cells (45, 46). This may be explained by the fact that cells such as ganglion cells that have a high density of mitochondria may have greater availability of the reductase enzymes required for its adduction (47, 48). However, in the context of the present study the localization of HP served to alert us to a possible connection between mitochondrial density and hypoxia inducible factors in retinal development.

We studied changes in the mitochondrial density of the developing retina until P9 and have shown a strong correlation between HIF-1α and HIF-2α nuclear localization in cells undergoing growth and increased mitochondrial biogenesis as functions of differentiation. This was particularly noticeable in developing horizontal cells (HZ) as although they are born and therefore committed to their fate during prenatal retinal development (E10-E16) (49), they only appear to undergo significant physical development during the postnatal period. This increase in size and generation of neural connections was associated with an increase in mitochondrial density between P3 and P5, and an even greater expansion in the period between P5 and P9. These changes coincided with strong expression and nuclear localization of HIF-1α and HIF-2α in the same period. HZ cells were also the first cells in the outer region of the NBL to show markedly increased expression and nuclear localization of AMPK carrying activating phosphorylations. AMPK represents a major signaling hub coordinating cellular nutrient and energy homeostasis (26, 50), including mitochondrial biogenesis *via* its direct regulation of PGC1α (51). In this regard, nuclear localization of PGC1α was prominent in ganglion and amacrine cells between P1 and P5, and HZ cells from P5. PGC1α has significant roles in cytoplasmic and intra-mitochondrial locations, and its typically punctate staining was present in the greater part of cytoplasmic regions in the neural retina, and in many instances could be found in bodies with the dimensions of mitochondria (52–54). Therefore, one or both HIF-1 isoforms, AMPK and PGC1α were consistently present in the nuclei of the most metabolically active cells (as judged by mitochondrial density) at the same stages of retinal development.

The above results were confirmed in secondary lens fiber development, apart from PGC1α which showed strong nuclear localization but only weak cytoplasmic staining in the fiber cells and was undetectable in the lens epithelium. This interesting difference between the lens and retina suggests more important non-transcriptional roles of the protein in neural tissue. Indeed, PGC1α is detected in both nuclei and cytoplasm of adult CNS neurons, where it is associated with neural activity (54), but shows stronger cytoplasmic staining during neural development in the postnatal period (55).

Weak nuclear staining for HIF-1α was detected in the lens epithelium and in the anterior aspect of the secondary fibers (data not shown) but nuclear localization was not observed. In comparison, HIF-2α showed similar cytoplasmic staining but also strong nuclear localization. As in the retina, it is logical that AMPK, PGC1α and HIF-2α should be resident in the same nuclei at the same stage in differentiating cells. Secondary lens fibers are extremely large crescent-shaped cells that differentiate from small epithelial cells in response to the high point of an ascending gradient of basic fibroblastic growth factor at the lens equator (56). The rapid change in cell volume and the accompanying need for increased protein synthesis (23) is clearly fueled by increased mitochondrial biogenesis, as demonstrated in the current study. It can be surmised that, as in the differentiation of retinal neurons, a growing energy deficit would be detected by AMPK that among its other responses would activate PGC1α to increase mitochondrial biogenesis (26, 51, 57).

The intriguing element in the progression of events described above, is the stabilization and nuclear translocation of HIF in concert with mitochondrial development. A basis for understanding this phenomenon is provided in a 2009 study by O’Hagan et al. showing that depletion of intracellular oxygen as a result of a PGC1α-mediated increase in mitochondrial biogenesis can stabilize HIF-1α under conditions where oxygen is limiting (25). These workers over-expressed PGC1α in primary human skeletal muscle myotubes and showed a resultant activation in a range of HIF-1 target genes. They went on to show that the HIF activation was caused by HIF-1α protein stabilization and not by transcriptional coactivation or other means. This work also demonstrated that increased mitochondrial biogenesis diverted oxygen from the oxygen-dependent HIF prolyl hydroxylases (58) that initiate the process leading to proteasomal destruction of HIF-1α (59, 60) and prevent its translocation to the nucleus. The authors emphasized that mitochondrial respiration can only impair other oxygen-dependent enzymes, such as HIF hydroxylases, when oxygen is barely sufficient and cannot be readily increased. This is similar to the situation in the developing retina and lens where the rapidly increasing energy demand of differentiating cells stimulates mitochondrial biogenesis. In the retina, a greater capacity for oxidative phosphorylation and oxygen consumption may create a relative oxygen deficit that can only be slowly met by HIF mediated angiogenesis that eventually provides more oxygen, enabling a further cycle of consumption and demand. This paradigm is congruent with the observed marker expression in secondary lens fiber development in the present study. However, the situation in the retina is more complex due to the widespread expression of HIF-1α in the pre-natal period (61). Kurihara and colleagues (61) showed that immediately following birth, nuclear HIF-1α declined throughout the retina except for cells corresponding to the ganglion cells and developing amacrine cells at the inner aspect of the NBL, as also shown in the present study. As ganglion and amacrine cells already had significant mitochondrial populations at P1, increasing mitochondrial activity in the low oxygen environment may simply have maintained HIF activation, rather than initiating it.

At P1 and P3, a similar pattern of nuclear localization was seen for HIF-1α and HIF-2α, although the latter showed greater expression and nuclear localization in the progenitor cells and much stronger staining in the horizontal cells at P1. This is logical as HIF-2α is stabilized at higher concentrations of oxygen than HIF-1α (62) and may not require the impact of mitochondrial oxygen usage for stabilization (25). Whether HIF-2α is present in the developing retinal neurons throughout the late prenatal and early postnatal periods, in the same way as HIF-1α (61) is not clear as the same study only showed positive staining for HIF-2α in the hyaloid vasculature, in spite of its normally strong expression in the astrocytes, shown in the present study and previously by Mowat et al. (10). HIF-2α expression has also been shown in retinal progenitor cells (8) and its genetic deletion has a severe impact on retinal vascular development (8).

Increased expression of HIF during differentiation and mitochondrial expansion appears counterintuitive when viewed from the established paradigm of its role in pathological hypoxia and physiological adaptation to reduced oxygen in a developed system (63, 64). However, the situation is radically different in developing cells with gradually increasing oxygen usage within an oxygen-poor environment. It should also be considered that a growing body of evidence supports the view that retinal neurons rely on aerobic glycolysis (65, 66). Although most of the work has been done on photoreceptor cells (67), there is evidence that some classes of amacrine cells support an active glycogen metabolism with high expression of glycogen phosphorylase in the normal retina and glycogen overload in diabetic retinopathy (31). Therefore, it could be speculated that HIF activity is necessary for aerobic glycolysis in developing inner retinal neurons, with its propensity to enable higher levels of biosynthesis than oxidative phosphorylation alone (65, 66).

In overview, at the most basic level the process of differentiation from a progenitor cell to a fully differentiated neuron entails external signals inducing a cascade of transcription factors that define the eventual fate of the cell (68). The implementation of the requisite genetic programs will then increase the energy intensive processes of protein and nucleic acid biosynthesis, membrane generation, intracellular transport, including consumption by motor proteins and construction of transport networks such as microtubules and actin fibers. Also, the energy cost of the intracellular signaling required to coordinate the above processes requires multiple phosphorylation-dephosphorylation cycles that consume huge amounts of ATP (23, 69). In the case of neurons, the vast energy cost of nerve conduction and neurotransmission can be added as soon as those processes commence (70–72). Therefore, it is plausible that increasing oxygen usage by an expanding population of mitochondria should invoke HIF in the manner described by O’Hagan et al. (25).

Nuclear localization of phospho-glycogen synthase (pGS) matched the same expression pattern as the other markers examined in the present study and correlated with increasing mitochondrial density in the retinal neurons. It appears that retinal progenitor cells rely on energy derived from aerobic glycolysis using glycogen as an intermediate and switch to oxidative phosphorylation after differentiation (17), which is an established pattern (73). Although that work was not performed in mice, the nuclear localization of phosphor-glycogen synthase (pGS) in the present study suggests that if an active glycogen metabolism exists in the post-natal retinal progenitors in the mouse, it is progressively suppressed in line with differentiation and increasing mitochondrial metabolism. Retinal neurons, in common with those of the brain have the necessary apparatus to synthesize and metabolize glycogen, but under normal circumstances glycogen synthase remains inactive through phosphorylation and nuclear sequestration (30, 31). This study has shown that by P9 the majority of retinal neurons and RPE cells had some pGS sequestered within their nuclei and that in adulthood the pattern had extended to almost all retinal neurons and photoreceptor cells, as previously shown in the rat (31).

In relation to retinal vascular development this study supports the conclusions of Usui et al. (7), that increased development of the horizontal and amacrine cells mediates the development of the deep and intermediate vascular plexuses and suggests that a determining factor in interneuron-mediated angiogenesis may be their high mitochondrial content. This study has also shown that the high mitochondrial density in the HZ cells matches the development and associated energy expenditure of their biosynthetic organelles, the RER and Golgi.

Also, in relation to retinal vascular development, it should be recognized that the primary retinal vascular plexus develops during the time period covered by the present study, completing between P7 and P8 (74, 75). Indeed, by P9 angiogenic capillaries from the primary plexus would have reached the outer plexiform layer and commenced the process of lateral expansion to develop the deep vascular plexus. The impact of the deep plexus may be responsible for the gradient of nuclear-localized HIF-1α observed in inner nuclear layer at P9 in the present study, with greater nuclear HIF already present in the amacrine cells, predicting their increased VEGF secretion that induces the intermediate vascular plexus as described by Usui et al. (7).

The ongoing development of the primary vascular plexus was also detectable in the reduced nuclear localization of HIF-1α in the ganglion cells at P9. The same was true, but to a lesser extent for HIF-2α which as mentioned above is more stable at higher oxygen levels (62). Future studies using retinal flat-mounts may be able to identify whether HIF expression in the ganglion cell layer is lower in the proximity of retinal arterioles than close to the venules as previously shown for VEGF expression by retinal astrocytes (76). This is quite conceivable as unpublished studies in our laboratory have shown that arterial oxygen not only inhibits endothelial cell survival in the periarteriolar capillary-free zones of the primary plexus but causes an obvious delay in the development of the intermediate plexus, and to a lesser extent the development of the deep plexus immediately beneath the arterial vessels.

In conclusion, we have provided evidence that cells located within a few microns of each other can have radically different responses to hypoxia, depending on their metabolism and mitochondrial activity. It appears that physiological hypoxia is a cell-specific phenomenon with different cells experiencing a range of oxygen deficits within a tissue with no spare oxygen capacity.

The current report appears to be the first demonstration of HIF-2α in the lens, although HIF-1α has been implicated in regulation of proliferation in age (77) and in fiber maturation (78). Likewise, AMPK and PGC1α do not appear to have been examined in lens development.

## Data Availability Statement

The original contributions presented in the study are included in the article/**Supplementary Material**. Further inquiries can be directed to the corresponding author.

## Ethics Statement

The animal study was reviewed and approved by Queen’s University Belfast, Animal Welfare and Ethical Review Body (AWERB).

## Author Contributions

TG: work planning, acquisition of eyes, confocal microscopy, original draft preparation. TB and NT: specimen processing, immunohistochemistry, confocal microscopy. DM: work planning, review and editing of manuscript. All authors contributed to the article and approved the submitted version.

## Conflict of Interest

The authors declare that the research was conducted in the absence of any commercial or financial relationships that could be construed as a potential conflict of interest.

## Publisher’s Note

All claims expressed in this article are solely those of the authors and do not necessarily represent those of their affiliated organizations, or those of the publisher, the editors and the reviewers. Any product that may be evaluated in this article, or claim that may be made by its manufacturer, is not guaranteed or endorsed by the publisher.
