# Supplementary material for: Markers of Hypoxia and Metabolism Correlate With Cell Differentiation in Retina and Lens Development
Source: Front Ophthalmol (Lausanne). 2022 Apr 22;2:867326. doi: 10.3389/fopht.2022.867326 (PMC11182328; doi:10.3389/fopht.2022.867326)
Supplement: Supplementary file 1 [file DataSheet_1.pdf]

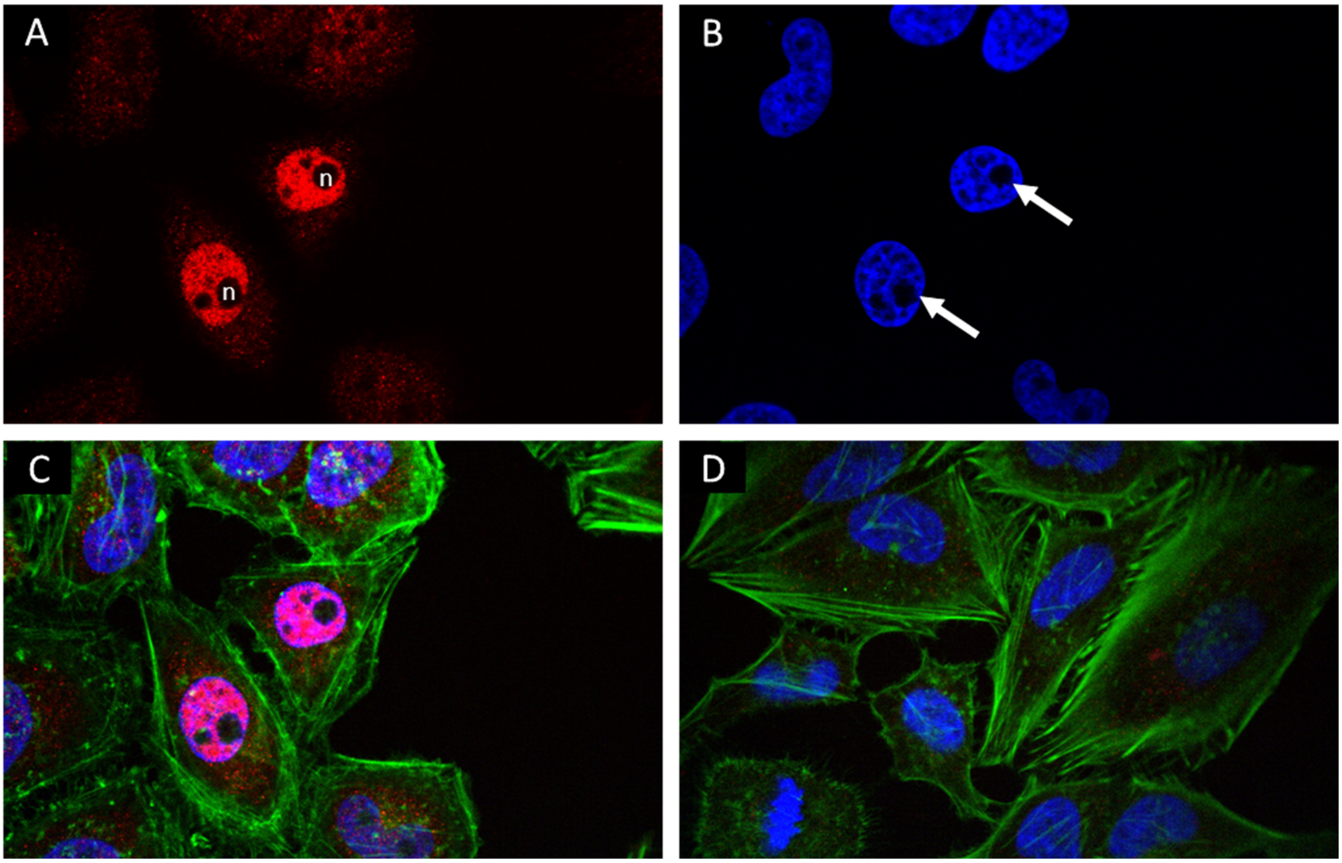

Fig-S1: Confocal micrographs showing Hif-1α staining in HeLa cells following a 24-hour exposure to 100 µM cobalt chloride. **(A):** The red channel image shows strong nuclear staining for HIF in 2 cells with prominent nucleoli (n). **(B):** The blue channel image shows staining of the DNA with DAPI (0.1µg/ml in PBS). The nucleoli remain unstained as DAPI does not stain RNA (arrows). **(C):** Colocalization of HIF and DNA indicates that the HIF is confined to the euchromatic regions of the nucleoplasm and excluded from the heterochromatin that stains intensely with DAPI. The cortical actin is stained green with FITC-phalloidin. **D:** Untreated cells showed no nuclear localization of Hif-1α

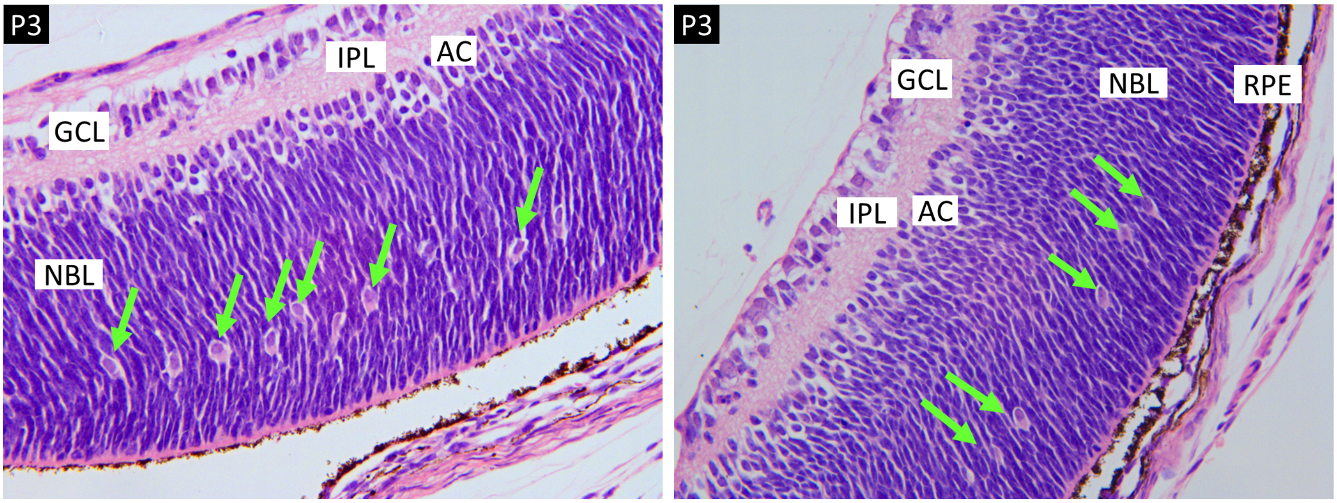

Fig-S2: Haematoxylin and eosin stained sections of P3 retina. Both images show the typical location of the developing horizontal cells (HZ- green arrows) in the outer third of the neuroblastic layer (NBL). The HZ cells have pale-stained round or oval nuclei and abundant cytoplasm in comparison to the darkly stained fusiform nuclei and scanty cytoplasm of the surrounding progenitor cells. The HZ cell nuclei are also polarised toward the inner retina with their cytoplasm facing the retinal pigment epithelium (RPE).
